# Rituximab Prevents Stroke Recurrences in Atypical Chronic Immune-Mediated Thrombotic Thrombocytopenic Purpura

**DOI:** 10.1055/s-0038-1676357

**Published:** 2018-12-05

**Authors:** Henry Dupuy, Estibaliz Lazaro, Irène Machelart, Jean-François Viallard, Paul Coppo, Etienne Rivière

**Affiliations:** 1 Department of Internal Medicine and Infectious Diseases, University Hospital Center of Bordeaux, Haut-Leveque Hospital, Pessac, France; 2 ImmunoconcEpT and FHU ACRONIM, UMR CNRS 5164, Bordeaux, France; 3 UMR CNRS 5164, ImmunoconcEpT and FHU ACRONIM, University of Bordeaux, Bordeaux, France; 4 Cardiovascular Adaptation to Ischemia, INSERM, Pessac, France; 5 Cardiovascular Adaptation to Ischemia, University of Bordeaux, Pessac, France; 6 Department of Hematology, Saint-Antoine Hospital, Paris, France; 7 Department of Hematology, University Pierre and Marie Curie (UPMC Paris 6), Paris, France; 8 French Referral Center for Thrombotic Microangiopathies, APHP, Paris, France

Thrombotic thrombocytopenic purpura (TTP) is a serious and rare disease diagnosed by <10% activity of disintegrin and metalloprotease with thrombospondin type-I repeats-13 (ADAMTS13). About 75% of cases are of immune-mediated origin (iTTP), with detectable anti-ADAMTS13 blocking autoantibodies. 1 2 Onset is usually sudden, with profound thrombocytopenia, microangiopathic hemolytic anemia, and multiple organ failures. Since plasma exchanges and steroids have been prescribed, patients' survival rate has increased to 85%. 3 4 Once remission is achieved, surveillance is based on the ADAMTS13 activity level, which is considered predictive of relapses. 5 Preventing potentially serious and sometimes atypical iTTP recurrences remains a major difficulty. In iTTP patients with persistent, severe, immune-mediated ADAMTS13 deficiencies, preemptive rituximab infusions allowed ADAMTS13 levels to rise and thereby avoid clinical relapses. 6 7 8 We report the unusual case of a patient who, 30 years after the initial episode, relapsed with idiopathic stroke and very mild cytopenias; several rituximab infusions achieved gradual recovery of ADAMTS13 activity and its inhibitory autoantibody gradually disappeared.

**Fig. 1:**
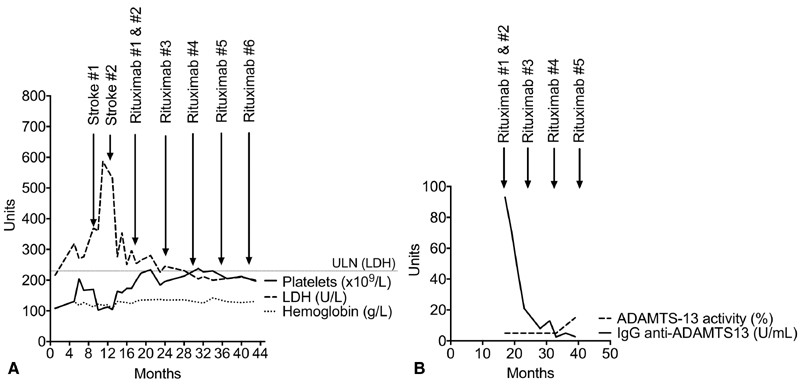
Evolution of this acquired immune-mediated thrombotic thrombocytopenic purpura. Patient's blood **(A)** and specific laboratory **(B)** parameters under rituximab starting in 2015. ADAMTS13, disintegrin and metalloprotease with thrombospondin type-I repeats-13; IgG, immunoglobulin G; LDH, serum lactate dehydrogenase; ULN, upper limit of normal.

In 1985, when he was 37 years old, the patient probably had his first known iTTP episode, with acute sensory and motor deficits and then coma, treated with steroids, platelet transfusions, aspirin, and dipyridamole. The outcome was favorable with no sequelae. Because iTTP was not diagnosed at that time, he had no specific medical follow-up. In 2014, when he was 66 years old, routine blood tests revealed anemia (hemoglobin, 109 g/L) and thrombocytopenia (108 × 10 ^9^ platelets/L). Bone-marrow aspirate showed features of refractory cytopenias with multilineage dysplasia and deletion of the long arm of chromosome 8, suggestive of low-risk myelodysplastic syndrome (International Prognostic Scoring System 0.5). During follow-up, the patient reported recurrent headaches and color vision disturbances. Monthly laboratory tests showed platelet counts fluctuating between 104 and 185 × 10 ^9^ /L, hemoglobin at 107 to 127 g/L, and lactate dehydrogenase (LDH) at twice the upper limit of normal (ULN). One year later (in 2015), when he was 67 years old, he suffered two multifocal ischemic strokes at a 3-month interval, with hemiparesis and dysarthria but progressive full recovery. Brain magnetic resonance imaging confirmed recent ischemic lesions and many older ones. The diagnostic work-up excluded an atheromatous origin and antiphospholipid syndrome. During the 3 months between his two hospitalizations, his platelet counts exceeded 100 × 10 ^9^ /L, with a platelet drop from 170 to 105 × 10 ^9^ /L between the two strokes; hemoglobin remained stable at 120 g/L and LDH rose to 590 U/L (3× ULN). Six months later, the patient was asymptomatic and laboratory test values were normal ( Fig. 1 ). At that time, iTTP was suspected, confirmed by <5% ADAMTS13 activity and high immunoglobulin G anti-ADAMTS13 at 93 IU/L (normal <15 IU/L). Although no frozen serum samples from previous hospitalizations were available to document severe ADAMTS13 deficiencies, our patient's whole history strongly suggests that his iTTP diagnosis could have been made in 1985. A strategy to prevent subsequent iTTP relapses was started in January 2016: rituximab (1 g), infused on days 1 and 15, then every 6 months. Before rituximab was prescribed, he had experienced three TTP recurrences (2014 and two in 2015). Over the next 2 years, he remained asymptomatic and usual laboratory parameters rapidly returned to normal ( Fig. 1A ). Notably, despite the documented rapid and persistent clearance of anti-ADAMTS13 autoantibodies after the first rituximab infusion ( Fig. 1B ), his ADAMTS13 activity did not rise until October 2017, nearly 2 years after the first rituximab infusion.

This patient represents an atypical case of chronic iTTP, with very mild cytopenias but recurrent strokes, stopped by prolonged rituximab use. The usual iTTP clinical picture is characterized by an acute and profound platelet count decrease to <30 × 10 ^9^ /L, which was not the case for our patient. Two points highlight its peculiarity: recurrent strokes with elevated LDH in our iTTP patient, who had been completely asymptomatic for 30 years, and his documented late recovery of ADAMTS13 activity, more than 1 year after rituximab cleared the anti-ADAMTS13 autoantibodies to prevent additional stroke recurrences.

First, the patient's very late iTTP recurrence without obvious cytopenias is very unusual. This clinical picture belongs to a subgroup of idiopathic ischemic strokes called embolic stroke of undetermined source (ESUS), which accounts for 25% of ischemic strokes, with no etiology retained after a standard diagnostic work-up. 9 Without any specific treatment, 4.5% of patients experience stroke recurrence at 1 year and 23% have a risk of death or disability at 6 months. 10 11 The main ESUS etiologies are rare cardioembolic diseases 12 13 and congenital or acquired thrombophilic states (myeloproliferative neoplasms, antiphospholipid syndrome, 13 paroxysmal nocturnal hemoglobinuria, heparin-induced thrombocytopenia, and TTP). 14 15 The chronic, insidious evolution of this patient's iTTP during the 30 years after the initial diagnosis raises questions and supports the hypothesis of managing iTTP as a chronic disease, rather than an acute one, with possible repeated flares, and suggests considering any idiopathic stroke with thrombocytopenia, even mild, potentially iTTP. 16 Notably, an LDH concentration >10 times above the ULN is a poor prognosis factor for iTTP, because it reflects organ damage. 17 18 However, in the absence of hemolysis, even a slightly higher abnormal LDH level can be the sign of persistent microthrombotic activity. Our patient's initial LDH concentration was markedly above normal without hemolysis and dramatically elevated LDH levels preceded the strokes. Importantly, his LDH level normalized under rituximab.

Second, his ADAMTS13 activity remained <10% for 2 years, despite four rituximab cycles, without any clinical or biological consequences. It should be noted that real ADAMTS13 activity may vary widely, even in a given patient, meaning it should be retested before every rituximab infusion. 19 However, ADAMTS13 activity remaining <10% following iTTP remission triples the relapse risk. 5 Indeed, severe ADAMTS13 deficiency is probably necessary to trigger an iTTP episode, but other factors can be incriminated, e.g., inflammation, endothelium dysfunction, or circulating DNA and histones. 18 19 20 In inflammatory states, with or without infection, human neutrophil peptides (HNPs) released from degranulated neutrophils can block von Willebrand factor (VWF) cleavage by ADAMTS13 in a concentration-dependent manner. HNPs can also trigger the secretion of platelet-granule contents, especially soluble CD154, and inhibit fibrinolysis, leading to a procoagulant state. 20 Changes of VWF secretion, multimer distributions, and plasma levels might also trigger TTP. 19 21 22 Some proteins might counterbalance ADAMTS13 deficiency, which might explain why our patient had no recurrent iTTP episodes between 1985 and 2014. Indeed, serine proteases, e.g., plasmin, thrombi or the leukocyte protease elastase, can also cleave VWF. Plasmin, too, has notable VWF-cleaving ability: during acute iTTP episodes, it cleaves VWF multimers in a shear-dependent manner. 23 24 Finally, physiological plasma high-density lipoprotein or apolipoprotein A1 concentrations inhibit shear-induced VWF self-association, thereby preventing platelet adhesion. 25

Without preventive treatment, 25 to 40% of patients with low ADAMTS13 activity will relapse after 1 year of follow-up. 26 27 Treating these patients with rituximab could limit the iTTP recurrence rate and increase the mean ADAMTS13 activity up to 46% at 3 months. 6 Jestin et al recently showed that preemptive rituximab prevented iTTP relapses in 85% of their patients, by inducing an ADAMTS13 conformational change in responsive patients. 8 They also suggested that depletion of B cells and short-lived plasmocytes secreting anti-ADAMTS13 autoantibodies might be the main mechanism of action of rituximab in iTTP patients. 28 However, despite rituximab, ADAMTS13 activity remains low in some patients.

In this setting, additional therapeutic pressure with new therapeutics, such as recombinant ADAMTS13 or caplacizumab, might be helpful. 29 30 Indeed, recombinant ADAMTS13 could be effective, acting just like endogenous ADAMTS13, as demonstrated by in vitro and in vivo studies, 29 although those results remain to be confirmed in phase II and III trials. In addition, caplacizumab, a humanized anti-VWF nanobody, rapidly blocks the interaction of VWF multimers with platelets, thereby inhibiting microthrombus formation and accumulation. In the TITAN study 31 on iTTP patients undergoing plasma exchanges, caplacizumab normalized platelet counts more rapidly than placebo, and recipients required fewer plasma exchanges and achieved a higher percentage of complete remissions. However, moderate bleeding-related adverse events were more frequent in the caplacizumab group. Authors of another recent study reported that caplacizumab-treated patients experienced fewer thromboembolic events and iTTP exacerbations. 30

In conclusion, this case illustrates the importance of suspecting iTTP, even without major thrombocytopenia, during any thrombotic event. It highlights iTTP heterogeneity and suggests considering it as a chronic disease with possible flares, even after long-term follow-up. Rituximab would seem to be the main treatment to prevent iTTP recurrences to improve survival of patients with this disease.
